# Supplementary material for: Complex‐centric proteome profiling by SEC‐SWATH‐MS
Source: Mol Syst Biol. 2019 Jan 14;15(1):e8438. doi: 10.15252/msb.20188438 (PMC6346213; doi:10.15252/msb.20188438)
Supplement: Supplementary file 7 — Dataset EV6 [file MSB-15-e8438-s007.zip › feature_plots_bioplex/Q96EF6.pdf]

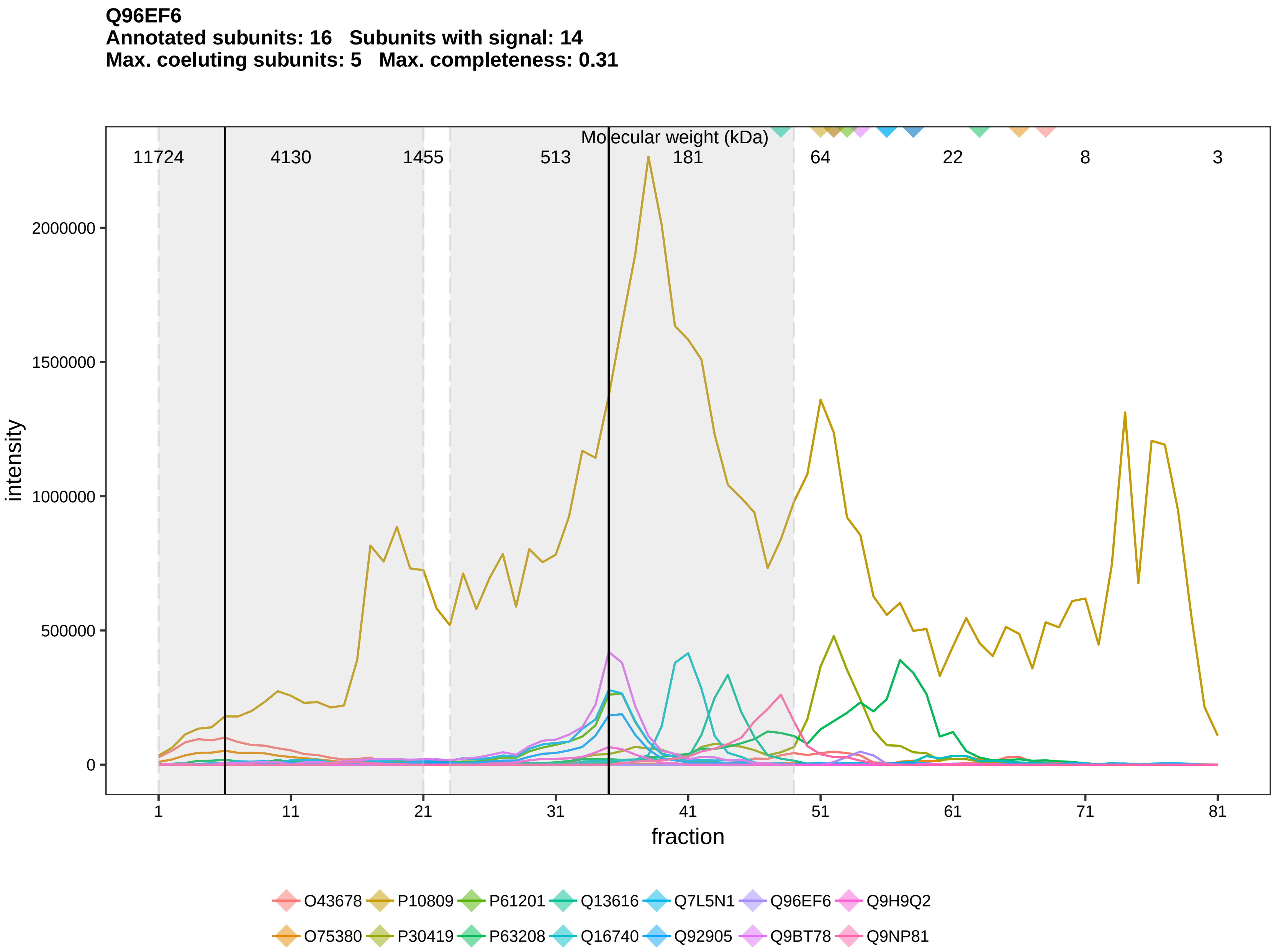

Q96EF6
Annotated subunits: 16 Subunits with signal: 14
Max. coeluting subunits: 5 Max. completeness: 0.31
Molecular weight (kDa)
11724
4130
1455
513
181
64
22
8
3
intensity
2000000
1500000
1000000
500000
0
fraction
1
11
21
31
41
51
61
71
81
O43678
P10809
P61201
Q13616
Q7L5N1
Q96EF6
Q9H9Q2
O75380
P30419
P63208
Q16740
Q92905
Q9BT78
Q9NP81
